# Non-typhoidal *Salmonella* in food animals in Paraguay: predominant serovars and resistance phenotypes

**DOI:** 10.3389/fvets.2025.1521469

**Published:** 2025-03-25

**Authors:** Rossana Irrazábal, María V. Iriarte, Julio Alvarez

**Affiliations:** ^1^Department of Epidemiologic Surveillance, National Animal Health and Quality Service (SENACSA), San Lorenzo, Paraguay; ^2^Faculty of Veterinary Medicine, University of Republic, Montevideo, Uruguay; ^3^VISAVET Health Surveillance Centre, Universidad Complutense, Madrid, Spain; ^4^Department of Animal Health, Faculty of Veterinary Medicine, Universidad Complutense, Madrid, Spain

**Keywords:** antimicrobial resistance, *Salmonella* Heidelberg, *Salmonella panama*, serovars, surveillance

## Abstract

Surveillance of antimicrobial resistance (AMR) in *Salmonella* in livestock (poultry, pig, and cattle) is crucial to maintain food safety. Given the lack of information on the situation in livestock in Paraguay, the aim of this study was to determine the most frequent *Salmonella* serovars in poultry, pig and cattle sampled in slaughterhouses in the country in 2020–22 along with their AMR phenotypes using data from a national pilot program. Out of 1,161 samples collected from slaughtered animals originating from 189 farms nationwide, *Salmonella* was isolated from 91/384 (23.7%) samples from poultry, 52/390 (13.3%) from pigs and 6/387 (1.6%) from cattle. Seven serovars were identified in poultry, with Heidelberg being the most frequent (82.4% of 91 isolates), while the most frequent serovars in pigs were Panama (48.1%) and Typhimurium (38.5%), and only two serovars (Cerro and Braenderup) were identified in cattle. The proportion of resistant isolates ranged from extremely high (70–83% for nalidixic acid and tetracycline) and high (25–40% for nitrofurantoin and ampicilin) to low-moderate (8–18% for cefixime, cefotaxime, amoxicillin, and trimethoprim- sulfamethoxazole) and very low-low (<6% for ciprofloxacin and gentamicin) depending on the antimicrobial. Up to 23 different resistance profiles were found, ranging from pansusceptible (18/143 isolates) to resistance to 2–7 antimicrobials (median = 2), with the predominant serovars in poultry and swine typically being resistant to ≥3 antimicrobials. These results should be backed-up with genomic analyses to determine the genetic mechanisms involved in the resistance profiles observed in order to support coordinated actions for AMR surveillance and control in the country.

## 1 Introduction

Salmonellosis continues to be a significant global public health concern, given the difficulties associated with its control (1). Preventing *Salmonella* infections can be challenging due in part to its complex epidemiology, with numerous serotypes exhibiting different host-preference ranges, patterns of transmission and levels of virulence (1, 2). The bacterial genus *Salmonella* is divided into two species, *Salmonella bongori* and *S. enterica*, and several subspecies, with *S. enterica* subsp. *enterica* being responsible for almost all *Salmonella* infections in warm-blooded animals. Within this subspecies there are over 2,500 serovars that differ in their distribution and their impact on their hosts, with only a few causing most infections in humans and domestic animals (3). Non-typhoidal *Salmonella* (NTS) serovars (those different from *S*. typhi and Paratyphi) cause foodborne infections associated to gastroenteritis and can have a broad host range involving food animals. Although a wide range of NTS serovars can cause disease in humans, *S.* enteritidis and *S.* typhimurium are the most frequently reported serovars and therefore the most relevant for public health (4, 5).

Multiple food animals can act as reservoirs of NTS, making them potential sources of infection for humans through the consumption of contaminated food. NTS stands as the second most prevalent cause of foodborne disease in Europe, only exceeded by *Campylobacter* (6). In 2010, the World Health Organization (WHO) estimated that there were approximately 153 million global infections of NTS, of which 56,969 resulted in fatalities, and almost half of the infections were attributed to contaminated food sources (7). Foodborne salmonellosis due to NTS is usually self-limited and usually does not require antimicrobials treatment. However, in some severe cases and/or those involving immunocompromised patients, the use of antimicrobials such as penicillin’s, cephalosporins and fluoroquinolones may be required (8, 9). However, the sometimes-unjustified use of antimicrobials in humans and animals has favored the selection and transference of resistance determinants in bacterial populations including *Salmonella* (10), thus potentially complicating treatment.

Certain countries such as the United States (US) and European Union (EU) member states have been implementing harmonized monitoring and control programs, which allow monitoring circulating NTS serovars and their antimicrobial resistance (AMR) phenotypes, for several years now (6, 11). In contrast, in South America there is still a lack of understanding of the epidemiological burden of NTS due to the absence of systematic large-scale surveillance in the region (12). Nevertheless, some countries in the region such as Argentina, Chile, Colombia and Brazil, have already implemented official control and surveillance programs for *Salmonella*, while others, such as Paraguay, are moving forward in the implementation process. In this country a pilot program to monitor the presence of foodborne pathogens (including *Salmonella*) and AMR in the food chain has been recently implemented. The first stage of this program consisted of a survey across the main production systems in the country (beef cattle, poultry, and swine) in 2020, 2021 and 2022, respectively. The aim of this study was to analyze the data collected through this pilot program in Paraguay by describing the frequency of isolation of *Salmonella* and the different NTS serovars retrieved from each animal species along with their resistance profiles to help in the design of a robust surveillance program, to assess the public health risk posed by the different production systems, and to support policy makers in the design of targeted programs for the control of *Salmonella* infection in food-producing animals.

## 2 Materials and methods

### 2.1 Study population

Paraguay is divided in two regions (Eastern and Western) by the Paraguay River, including 39 and 61% of the country area, respectively. The most important livestock species in the country is cattle, with approximately 14 million heads in 137,610 farms, which are mainly (90%) beef farms. Cattle farming is distributed throughout the country, with approximately 47% of all cattle in the Western region (Chaco) and the remaining 53% in the more developed Eastern region. Other livestock populations in the country include poultry (267 industrial poultry farms with approximately 25.8 million poultry, and 50,000 backyard poultry farms with approximately 3.2 million animals) and swine (46,188 farms with around 1.5 million heads). Most poultry and swine farms are located in the Oriental region (SENACSA, unpublished).

### 2.2 Data source

This study was based on the data collected during a pilot program of AMR Surveillance in Paraguay. This pilot program, named National Integrated Monitoring System for AMR in the agrifood chain (SINMRA), consisted of a three-year sampling (from 2020 to 2022) focused on one animal species each year: cattle in 2020, poultry in 2021 and swine in 2022.

The sampling process was carried out at exporting slaughterhouses, which were selected according to its levels of slaughter. The selection of slaughterhouses was based on specific criteria including being certified industrial slaughterhouses registered with SENACSA and supervised by veterinary staff in which slaughter of animals and deboning of carcasses were carried out as part of the slaughtering activities. Among those, only those with slaughter volumes of cattle, pigs and poultry representing at least 6.5% of the national slaughter total capacity of the country were considered to ensure the inclusion of slaughterhouses with a significant activity. Each sampled slaughterhouse only culled one livestock species.

Cattle slaughterhouses selected for this monitoring represented 61% of the total of slaughtered cattle in Paraguay, while poultry and swine slaughterhouses represented 48 and 43% of the total poultry and pig slaughtered, respectively. Briefly, fecal samples from the cecum were collected for slaughtered cattle and swine by making a cut of 8–10 cm and extracting at least 30 mL of fecal content, which was placed in a plastic collection container using a wooden tongue depressor. For poultry, the whole cecum was collected from slaughtered animals. All samples were then placed in an isothermal container with cooling gels and transported immediately to the laboratory at a temperature between 0 and 15°C.

The sampling design and the number of samples was determined according to the recommendations in the *Terrestrial Animal Health Code*.^1^ A 50% individual prevalence was assumed for all three animal populations given the lack of reliable estimates on the expected prevalence, and the sample size was set assuming a 95% confidence level, resulting in 384 samples for each production system (beef cattle, poultry and swine). However, between one and 76 animals from a given farm were collected depending on the host species (see results). The online tool OpenEpi (13) was used for these calculations.

The annual sampling plan spread over a space of 6 months, and the frequency and the number of samples was determined for each slaughterhouse based on their slaughter capacity. Animals were selected at random from those present in the slaughterhouse on the day of sampling by the Official Veterinary Inspector.

### 2.3 Laboratory methods

The cecal samples collected at the slaughterhouses were sent to the laboratory of the National Animal Health and Quality Service of Paraguay (SENACSA) for *Salmonella* isolation according to the protocol outlined in the SINMRA resolution 820/2019. Briefly, four grams of fecal sample were mixed with tetrathionate broth and incubated for 18–24 h. Bacterial growth was then inoculated on Rappaport-Vassiliadis broth, incubated and recultured in XLD plates. Suspected *Salmonella* colonies were then inoculated onto McConkey plates and their identity confirmed using TSI and urease biochemical tests.

All *Salmonella* isolates retrieved were sent to the Central Public Health Laboratory (LCSP) for confirmation of their identity and their resistance phenotypes and for serotyping using the White-Kauffmann-Le Minor scheme. The antimicrobial susceptibility profile of all *Salmonella* isolates retrieved was determined at the SENACSA laboratory using the Kirby-Bauer disk diffusion technique. Based on the size (diameter) of the inhibition zones, isolates were classified as Sensitive (S), Intermediate (I) or Resistant (R) using the Clinical and Laboratory Standards Institute (14) clinical breakpoints (15) (Table 1). Antimicrobial susceptibility testing was performed considering the following 10 antimicrobials and concentrations per disk as established in the SINMRA: nalidixic acid (NAL, 10 μg), amoxicillin (AMX, 30 μg), ampicillin (AMP, 10 μg), cefixime (CFM, 5 μg), cefotaxime (CTX, 30 μg), ciprofloxacin (CIP, 5 μg), gentamicin (GEN, 10 μg), nitrofurantoin (NIT, 300 μg), tetracycline (TET, 30 μg), and trimethoprim-sulfamethoxazole (SXT, 1.25/23.75 μg).

**Table 1:** Percentage of isolates resistant to each antimicrobial according to the species of origin and clinical breakpoint used and presence of significant differences between species (data from cattle not shown since all isolates were pansusceptible).

Antimicrobial	IZD breakpoints^a^ (mm)	% Total (*n* = 149)	% Poultry (*n* = 91)	% Swine (*n* = 52)	Fisher exact test
Tetracycline (TET)	≤ 11	83.9	89.0	84.6	0.62
Nalidixic acid (NAL)	≤ 13	70.5	**89.0**	**46.2**	**< 0.001**
Ampicillin (AMP)	≤ 13	38.3	**12.1**	**88.5**	**< 0.001**
Nitrofurantoin (NIT)	≤ 14	25.5	28.6	23.1	0.62
Amoxicillin (AMX)	≤ 13	18.1	**11.0**	**32.7**	**0.009**
Trimetoprim–Sulfamethoxazole (SXT)	≤ 10	8.7	7.7	11.8^b^	0.62
Cefixime (CFM)	≤ 14	8	12.1	2.1^c^	0.098
Cefotaxime (CTX)	≤ 19	8	12.1	1.9	0.098
Gentamicin (GEN)	≤ 12	5.4	**1.1**	**13.5**	**0.009**
Ciprofloxacin (CIP)	≤ 15	4.7	5.5	3.8	1.00

a Inhibition zone diameters below which isolates were classified as resistant.

b 51 isolates tested.

c 47 isolates tested.

### 2.4 Data analysis

All information on the isolates provided by SENACSA Department of Epidemiology and the SENACSA Laboratory of SENACSA (code of the farm of origin of the sampled animal, location of the farm, culture result and serovar and antimicrobial susceptibility profile in case of isolation of *Salmonella*) was recorded in a database in Excel.

For the geographical visualization of the farms from which the sampled animals originated, coordinates describing the location of the farms collected in Excel were imported into ArcGIS version 10.5 (ESRI^®^) software and mapped using the WGS 1984 coordinate reference system.

Additionally, we performed a spatial analysis to detect clusters of farms from each animal species in which *Salmonella* spp. was isolated using the Bernoulli model of the spatial scan statistic, implemented using SatScan V9.6^®^ (16).

Resistance levels for each antimicrobial were classified as ‘rare’: <0.1%, ‘very low’: 0.1–1.0%, ‘low’: >1.0–10.0%, ‘moderate’: >10.0–20.0%, ‘high’: > 20.0–50.0%, ‘very high’: > 50.0–70.0% or ‘extremely high’: > 70.0% according to previous recommendations (17). Information on the resistance phenotype of the isolates (S, I or R) according to the CLSI clinical breakpoints were compared using the Fisher’s exact test to assess differences in the proportion of isolates that were classified as resistant (R) to a given antimicrobial depending on the host species adjusting by multiple comparisons through the Benjamini-Hochberg procedure (18). In addition, the Kruskal-Wallis test, followed by a *post hoc* test (Dunn’s) with correction for multiple comparisons by Holm’s method (1979), was used to compare the number of antimicrobials to which isolates belonging to a given serovar or retrieved from a certain host species were classified as resistant. These statistical analyses were performed in RStudio version 4.2.2 using the FSA package (19). Finally, resistotypes, consisting in the concatenation of the susceptible/intermediate or resistant value for the 10 antimicrobials were also recorded and compared depending on host species/serovar. The proportion of multidrug resistant (MDR) isolates, defined as those resistant to one or more antimicrobials in three or more antimicrobial classes, was also calculated per host and serovar.

## 3 Results

### 3.1 *Salmonella* spp. detection and serotyping

**Figure 1:**
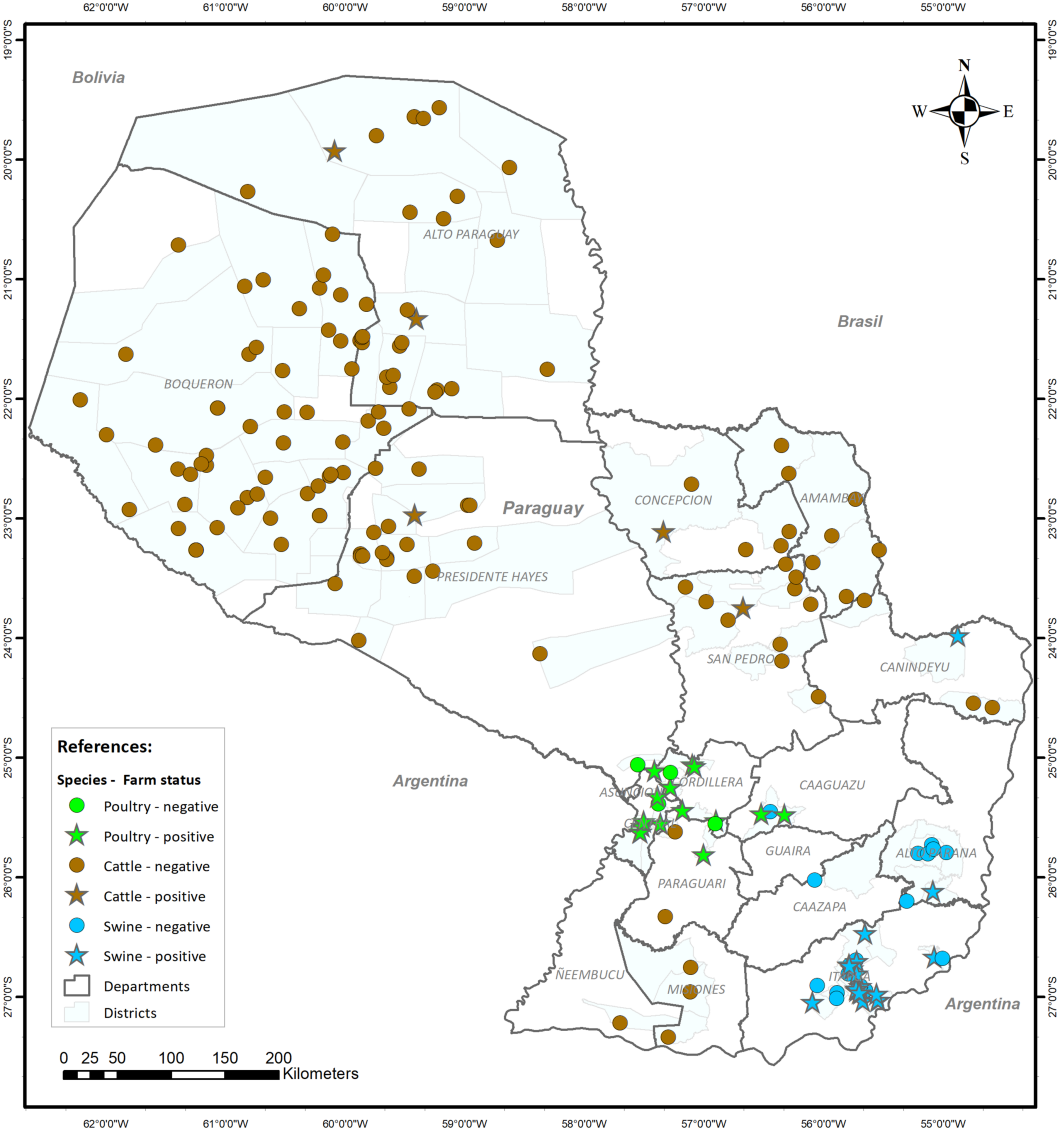
Geographical distribution of the 189 farms sampled by species and whether *Salmonella* spp. was retrieved from animals from each farm.

Figure 1 presents the geographical distribution of farms of different species that were sampled during the pilot program of AMR Surveillance in Paraguay. A total of 1,161 samples were collected, of which 384 (33.1%) were from poultry, 390 (33.6%) from swine, and 387 (33.3%) from cattle.

Even though initially the objective was to collect a single animal per batch (representing a single farm), the number of samples collected per premise varied depending on the host species. A total of 19 poultry farms were sampled, with a minimum of 17 and a maximum of 36 samples per farm (mean of 20 samples/farm), of which 78.9% had one or more samples positive for *Salmonella* spp. (91 individual positive samples; Table 2).

**Table 2:** Number of farms, samples collected by species and positive results for the presence of *Salmonella* spp.

Species	Samples		Farms	
	No of samples	Positive %	No of farms	Positive %
Poultry	**384**	91 (23.7)	**19**	15 (78.9)
Swine	**390**	52 (13.3)	**37**	19 (51.3)
Cattle	**387**	6 (1.6)	**133**	5 (3.7)
Total	**1,161**	**149 (12.8)**	**189**	**39 (20.6)**

For swine, 37 farms were sampled, with a minimum of one and a maximum of 76 samples per farm (mean of 10 samples/farm). *Salmonella* spp. was retrieved from approximately half of the farms (52 positive samples; Table 2).

Finally, a total of 133 cattle farms were sampled, with a minimum of 1 and maximum of 16 samples (mean of 3 samples/farm). Only five farms (and six samples) were positive for *Salmonella* spp. (Table 2).

No significant clusters of positive farms were identified when analyzing samples from each species separately. However, when all species were considered together, a significant cluster (*p*-value<0.0001) with a 287.4 radius (km) and encompassing all poultry and swine farms was found (data not shown), likely reflecting the spatial pattern in the distribution of the farms for these two species (located in the same region of the country) rather than clustering of positive farms.

#### 3.1.1 Poultry

Among the 91 isolates retrieved from poultry the following seven serovars were found: Heidelberg (75 isolates, 82.4%), Alachua (five isolates, 5.5%), Sandiego, Anatum and Tennessee (three isolates each, 3.3%), and Newport and Javiana (one isolate each, 1.1%).

*Salmonella* Heidelberg was also the predominant serovar at the farm level, and was found in 12 of the 15 positive farms. A single serovar was retrieved in 13 of these farms irrespective of the number of positive samples found, with the remaining two farms presenting four (Heidelberg, Anatum, Sandiego and Javiana) and two serovars (Heidelberg and Alachua).

#### 3.1.2 Swine

The 52 isolates found in swine were identified as Panama (25 isolates, 48.1%), Typhimurium (20 isolates, 38.5%), Derby (two isolates, 3.8%) and Anatum (two isolates, 3.8%), while another three isolates (5.8%) were only identified at the serogroup level as belonging to serogroup 7 (based on the “O” antigen of the bacterial lipopolysaccharide).

At the farm level the most abundant serovars were Typhimurium (present in 11 farms) and Panama (10 farms). In seven of the 19 positive farms for *Salmonella* spp., two serovars were identified while three serovars were found in one farm.

#### 3.1.3 Cattle

The six isolates were identified as *Salmonella* Braenderup (three isolates), and *Salmonella* Cerro (the remaining three isolates). Only one serovar was found in each of the five positive cattle farms.

### 3.2 Resistance analysis

The resistance phenotype of the 149 *Salmonella* isolates was determined for all 10 antimicrobials except for trimethoprim-sulfamethoxazole (one strain from swine not tested) and cefixime (five swine isolates not tested; Table 1; Supplementary Table S1).

Overall, when considering all *Salmonella* isolates irrespective of the host species from which they were recovered (*n* = 149), the highest resistance levels (>70%, considering only isolates in the “resistant” category according to the clinical CLSI breakpoints) were observed for tetracycline and nalidixic acid, followed by ampicillin (38.3%), nitrofurantoin (25.5%), and amoxicillin (18.1%), while resistance was below 9 % for the remaining antimicrobials (Table 1). When considering the host species from which isolates were retrieved, similar values were obtained for tetracycline, nitrofurantoin, trimethoprim-sulfamethoxazole and ciprofloxacin, while significant differences in resistance levels to nalidixic acid, ampicillin, amoxicillin, and gentamicin were observed: a significantly (*p* < 0.0001) higher proportion of isolates resistant to beta-lactams (ampicillin and amoxicillin) and aminoglycosides (gentamicin) were retrieved from swine compared to poultry, while the opposite was observed for nalidixic acid (Table 1). In addition, a borderline significantly difference (*p* = 0.098) between the proportions of isolates resistant to third generation cephalosporins depending on the host were also observed (with higher proportions in poultry isolates; Table 1). The proportion of isolates in the intermediate category for certain antimicrobials was also different depending on the host (and the serovar), with between one third and half of the isolates from swine classified as intermediate for nalidixic acid and amoxicillin compared to 0–1 isolate from poultry, while the opposite was true for ciprofloxacin (38.4% of intermediate isolates from poultry vs. only one from swine; Supplementary Table S1). The six isolates from cattle were all pansusceptible.

### 3.3 Resistotype analysis

**Figure 2:**
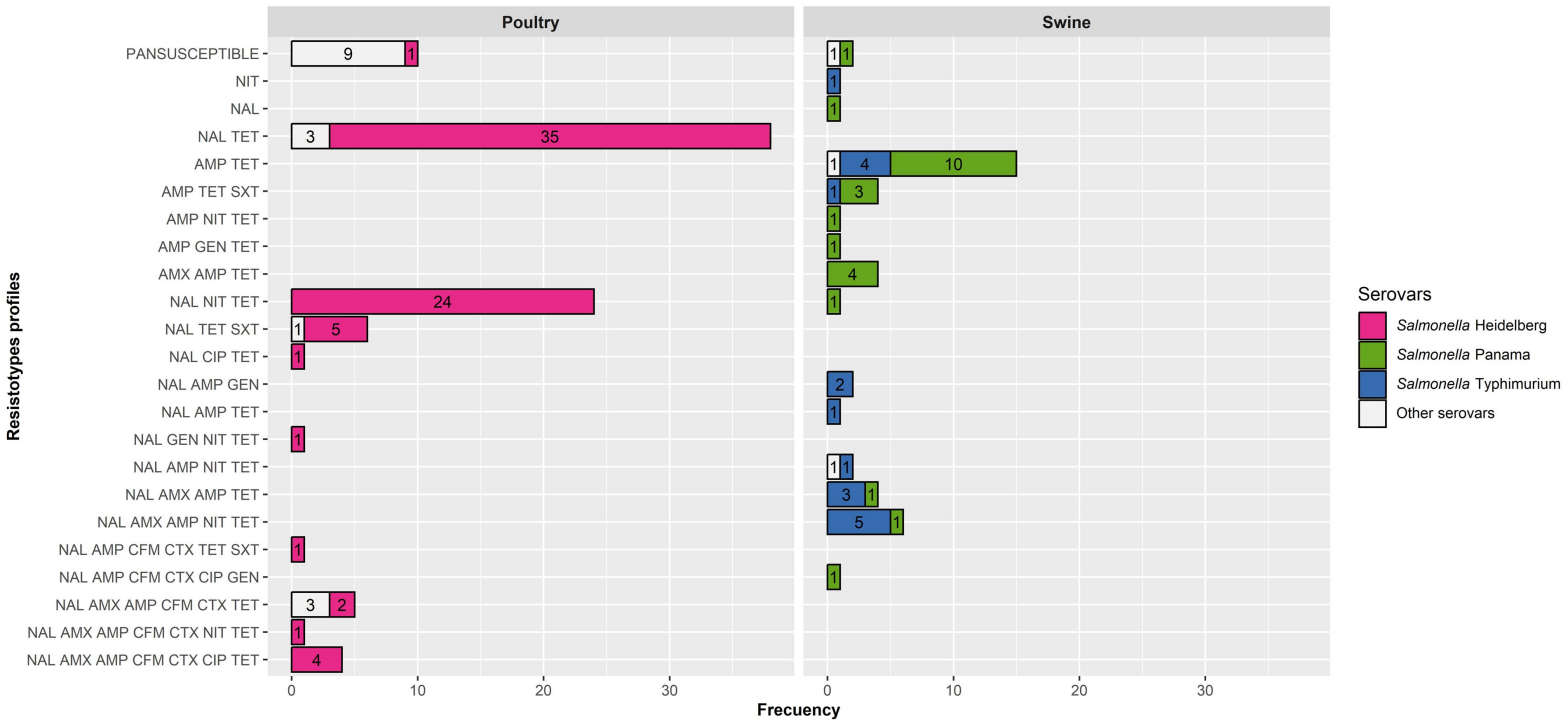
Resistotypes of the poultry and swine *Salmonella* isolates for those tested with 10 antimicrobials (91 from poultry and 46 from swine) per host and serovar.

Only isolates tested with all 10 antimicrobials (*n* = 143) were kept in this analysis. A total of 23 different resistotypes were found, involving from no resistance (pansusceptible resistotype, present in 18 isolates including all cattle isolates) to resistance to a range of 1–7 antimicrobials (median = 2, interquartile range = 2–3). Resistotypes for swine and cattle isolates are shown in Figure 2. Considering the three most frequent serovars (Heidelberg, Panama, and Typhimurium) no significant differences (*p* = 0.070) between the number of antimicrobials to which an isolate was resistant and its serovar were observed.

Ten resistotypes were identified among *Salmonella* Heidelberg isolates, with the most frequent ones involving nalidixic acid and tetracycline alone or plus nitrofurantoin (Figure 2). Nevertheless, eight isolates (retrieved from three farms) were resistant to 6–7 antimicrobials, including third-generation cephalosporins (Figure 2). Resistotypes in *Salmonella* Panama and Typhimurium isolates mostly comprised two to five antimicrobials, typically including resistance to ampicillin and tetracycline alone or plus other antimicrobials, but were distributed among eight and 10 different resistotypes, respectively. Resistance to over five antimicrobials was only found for isolates in the three predominant serovars (Heidelberg, Panama and Typhimurium) except for three *S*. Anatum isolates retrieved from the same poultry farms. The proportion of MDR isolates was very similar for poultry and swine (46.2 and 44.2%, respectively), with poultry MDR isolates belonging to the Heidelberg (*n* = 38), Anatum (*n* = 3) and Sandiego (*n* = 1) serovars, while the MDR swine isolates were Typhimurium (*n* = 13), Panama (*n* = 9) and the one identified up to the serogroup level as O7 (*n* = 1).

## 4 Discussion

*Salmonella* is one of the main foodborne zoonosis worldwide and, due to its zoonotic nature and the importance of the emergence of new resistant clones (20–23), monitoring programs have been implemented in multiple countries in order to assess the distribution and resistance phenotypes of the most common serovars in food animals (6, 11, 24–29). This study presents the first analyses of the pilot phase recently implemented in Paraguay, which seeks to describe the different *Salmonella* serovars that are present in the most important livestock species in the country (poultry, swine and cattle) and their antimicrobial resistance profiles. Our results indicate *Salmonella* differ in terms of their frequency, predominant serovars and resistance profiles in these production systems.

Out of the 19 poultry farms included in this study, 78.9% were positive for *Salmonella* spp., while the proportion of positive farms were 51.3% of the 37 swine farms and 3.7% of the 133 cattle farms. The higher prevalence of infection in swine and poultry compared with cattle is not surprising, since these two animal species are the ones classically associated with foodborne salmonellosis (30, 31). Nevertheless, the higher number of samples collected in pig and broiler farms compared with cattle could also result in a higher sensitivity at the farm level, thus leading to an artificially low prevalence in the latter species. Still, interpretation of our results at the farm level should be done carefully, since some of the positive animals at slaughter could have been infected after leaving the farm (during the transport or lairage stages) if in contact with shedders from different origins (32).

Differences in terms of the serovars found in each of the host species could be related to the different management practices (typically extensive/semi-extensive for cattle and intensive in the case of poultry and swine) and the lack of contact between farms housing different livestock species, coupled with the previously reported association between certain serovars and livestock species (e.g., Heidelberg in poultry or Typhimurium in swine) (33).

In our study, *S*. Heidelberg was the most frequent serovar in poultry samples (82.4% of the 91 strains isolated). This serovar was also among the most common in poultry samples from other countries in South America like Brazil (representing 39% of 98 isolates from chicken meat) (34) and Venezuela (31% of 77 strains isolated from poultry) (35). Nevertheless, other studies reported much lower frequencies among poultry *Salmonella* isolates (e.g., 5.5% of 280 strains from broiler chicken and turkey carcasses in Brazil) (36) and 16 of 133 isolates from a poultry farm in Chile (37). In Asian countries, such as Japan, *S.* Heidelberg was also among the most prevalent serovars, particularly in chicken meat (38). Other serovars that are commonly reported as very common in poultry in other countries in South America, North America or Europe such as *S.* Kentucky, *S*. enteritidis and *S.* infantis were not found in this study (11, 39, 40).

In pigs the most frequent serovar was *Salmonella* Panama (48.1% of the 52 strains isolated). This serovar., first described by E. O. Jordan in 1934 during the investigation of a foodborne infection in soldiers in Panama (41), has received limited attention in the past but is a major cause of human infection in areas of America and Asia, and has been linked to the pig industry in the past (42). Its presence had been previously reported in Brazil, where it was among the top serovars found in swine finishing herds (43) and in pigs sampled in the slaughterhouse (44). Similarly, it was the third most common serovar isolated from samples collected in in swine slaughterhouses in Argentina after *S*. Anatum and *S.* typhimurium (45). Typhimurium was the second most frequent serovar in our study (38.5% of the 52 isolates in swine), followed by serovars Anatum and Derby at very low frequencies, while these two serovars are the most frequent ones found in swine in the US according to the National AMR surveillance system (11).

The high frequency of *S.* typhimurium among pig isolates in our study is not surprising given that other countries reported this serovar as the most prevalent in this animal species. For example, *S.* typhimurium was reported in intensive and backyard swine production in Argentina between 2012 and 2018 (21.2% of 59 isolates) (46), and in Brazil this serovar was also found in pig slaughterhouses in 50.7% of 1,158 isolates (43). According to the last EFSA report for European countries, Typhimurium is also among the most frequent serovars reported in swine (4) and has been identified as a prevalent serovar in various regions of Asia. For example, in Japan, an analysis of 6,771 fecal samples from pigs across 73 farms identified *S.* typhimurium as the second most common serovar (47). In China, it accounted for 13.0% of 155 isolates in pork in Xuzhou (48) and was among the predominant serovars from pig slaughterhouses in Wuhan (49). In a different region of China (Shandong) two studies reported its prevalence: one identified *S.* typhimurium as the third most common serovar (11.4%) in food animals (50). while in the other found it was the second most prevalent serovar (32.0%) in meat processors (51). Additionally, in Sichuan, it was among the five most prevalent serovars, though primarily isolated from waterfowl (52). In Central Vietnam, it accounted for 12% of 99 strains in pig and poultry farms (53). The importance of *S.* typhimurium for public health is clear since it is one of the most frequent serovars found in clinical cases in multiple countries (2, 54), and was among the most prevalent serovars in foodborne outbreaks with a high rate of hospitalization in Paraguay (55).

Of the six isolates from cattle, three were identified as *Salmonella* Braenderup and three as *Salmonella* Cerro. These results differ from the first report on AMR in *S. enterica* in dairy farms in Uruguay, in which *Salmonella* Typhimurium was the most frequent serovar reported, followed by *S.* Dublin and *S.* Anatum, though this study was based on calves (some of them with clinical signs of salmonellosis) and thus could represent a different epidemiological situation to the one considered here (healthy animals entering the food chain) (56). On the other hand, *S.* Cerro was one of the most frequent serovars reported in bovine in United States (11).

No statistically significant clusters of positive farms were found in this study. However, when performing the analysis considering all species together, a spatial cluster was found. A possible explanation for this might be that most of the poultry and swine farms in Paraguay are concentrated in that area, in contrast to cattle farms that are spread throughout the country and would therefore not be related to the distribution pattern of *Salmonella*-positive farms.

In this study, the serovars with the highest levels of resistance were Heidelberg, Typhimurium and Panama. A previous study carried out in Paraguay by Ortiz et al. (63), showed that Heidelberg, Schwarzengrund and Typhimurium strains isolated from human and food samples presented the highest levels of resistance to clinically important antimicrobials. The high level of antimicrobial resistance of *S.* Heidelberg isolates is consistent with results reported by the Canadian Integrated AMR Surveillance (CIPARS) program, where 57.5% of the isolates were resistant to amoxicillin-clavulanic acid and ampicillin, and also to cephalosporins such as cefoxitin, ceftiofur and ceftriaxone (57).

The highest levels of resistance in this study were found against tetracyclines (89% in poultry and 84.6% in swine) and nalidixic acid (89% in poultry and 46.1% in swine). These findings are consistent with a study conducted in chicken isolates in Paraguay, of which more than 70% were resistant to nalidixic acid while all were susceptible to ciprofloxacin (also here the proportion of isolates resistant to ciprofloxacin was low—5.5%—in poultry isolates) (58). These findings agree with reports from Asian countries such as Thailand, China, and Vietnam, where high levels of resistance to tetracycline have been observed (53, 59, 60). Notably, in Thailand, isolates resistant to nalidixic acid were also reported, a result consistent with our studies (59). Quinolones were also the antimicrobial class against which swine isolates in Argentina were most frequently resistant, albeit at a lower level (24%) and also involving resistance to nalidixic acid (and susceptibility to ciprofloxacin) (45). Even though no differences in the proportion of isolates resistant to ciprofloxacin depending on the host were observed here, if isolates with an intermediate phenotype are considered our results suggest a much higher proportion of isolates with a non-wild type phenotype in poultry vs. swine, what would be in agreement with results from monitoring programs in Europe (where epidemiological cut-offs instead of clinical breakpoints are used) and the US, in which resistance to quinolones in general is much more frequent among broiler isolates (>30%) compared with those from swine (<5%) even though the predominant serovars may not be the same ones as in Paraguay (6, 11). Additional studies would be needed to determine whether the resistance mechanisms explaining these different phenotypes in poultry isolates in other regions are also present in Paraguay.

Beta-lactams (penicillin’s) were the next most frequent antimicrobial to which the isolates were resistant, although the level of resistance varied by antimicrobial and host species. Resistance to amoxicillin and ampicillin was significantly higher in swine compared to poultry (Table 1). However, in Thailand, the most frequent antimicrobial to which poultry isolates were resistant was ampicillin (34.2%) (59). Nevertheless, resistance levels to cephalosporins (cefixime and cefotaxime) were somewhat higher (12.1%) than in other countries including EU member states, where resistance to third generation cephalosporins was below 1% (6), or the United States (<5%) (11).

In terms of resistance to tetracyclines, we observed high resistance levels in poultry and swine (89.0 and 84.6%, respectively). These results are in agreement with two studies carried out in Argentina, in which resistance levels higher than 80% were reported in swine (45) and poultry isolates (61).

In our study, the six isolates form cattle were pansusceptible. In contrast, a high level of resistance to tetracycline (87.8%) was described in cattle isolates in Uruguay (56).

Multidrug resistance was observed in a very similar level among *Salmonella* isolates retrieved from poultry and swine, with one specific serovar accounting for most MDR isolates in poultry (Heidelberg, 92.9% of all MDR isolates) and, to a lower extent, swine (Typhimurium, 56.5%; Figure 2). Additional studies should be performed to evaluate the linkage between the resistance to some of these antimicrobials, ideally incorporating whole genome sequencing-based analyses to assess the genes involved in the resistance profiles observed in Paraguay. Nevertheless, the antimicrobial resistance profiles identified here have significant implications for public health since they can limit the effectiveness of certain treatments in the case of invasive infections (fluoroquinolones, cephalosporins) (62). Furthermore, AMR can also have an impact in animal production due to treatment failures, reduced productivity, economic losses, and an increased risk of zoonotic transmission through the food chain.

We acknowledge some limitations. First, our study only included exporting slaughterhouses which receive animals mainly from large size farms. These farms typically have higher levels of biosecurity compared with smallholder farms, and thus our results could represent an underestimation of the true prevalence in smaller livestock farms in the country. Therefore, the results cannot be extrapolated to the whole population of livestock in Paraguay. In future stages of SINMRA-Py, the study design should include local and municipal slaughterhouses to be able to reliably estimate the prevalence of *Salmonella* spp. in food animals in Paraguay. Furthermore, focusing the sampling on a different host species each year may hamper the comparison of results obtained in the three livestock species sampled if factors leading to a change in the prevalence of infection with specific serovars/resistance phenotypes in the short term were present. Therefore, if such factors were expected (due to, e.g., changes in policies regulating antimicrobial usage in livestock), including several species every year should be considered.

Second, some serotyping data were incomplete due to time and resource limitations. As a result, three isolates (5.8%) could only be identified at the serogroup level. While a complete serotyping would be ideal, given the low number of isolates that were not fully serotyped the results obtained provide valuable insights on the distribution of the predominant serovars across different host species.

Third, the staff collecting the samples were not aware about the origin of each animal, resulting in the collection of multiple samples from the same farm, especially for poultry and swine, in contrast to the original sampling strategy which assumed samples were independent. While this limits our ability to infer information at the farm level and therefore at the country level, it allowed to assess the within-farm variability, particularly for poultry, since a median of 20 samples were collected. On the other hand, a greater number of cattle farms were sampled with a low number of samples in each farm (mean of 2 samples). It seems possible that this hampered the accuracy in *Salmonella* detection in cattle farms. Furthermore, some of the infected animals could have been infected after leaving the farm during transport or lairage, particularly in the cases in which a single positive animal was detected even though ≥10 was sampled (3/15 positive poultry farm and 4/19 positive swine farms).

Finally, comparison of our results with those obtained in other studies should be done carefully due to possible differences in laboratory protocols used for *Salmonella* isolation. The use of internationally accepted protocols (e.g., the international ISO standard 6,579–1; 2017, which incorporates a pre-enrichment step in a non-selective liquid medium and a second selective plating medium in addition to the RVS broth) in the future could increase the external validity of our findings.

Nonetheless, our results demonstrate the presence of *Salmonella* spp. in food animals in Paraguay, entering the food chain through a pilot sampling performed with a national scope with a high prevalence in poultry and swine, which highlights the need to develop and implement a national *Salmonella* control plan. The identification of antimicrobial-resistant strains also underscores the critical necessity for collaborative efforts in the realms of production, food safety, and human health, as well as the need for ad-hoc programs to establish the baseline prevalence of *Salmonella* infection particularly in poultry and swine farms and transition the current SINMRA-Py pilot plan into a full-fledged national program that also includes human data. This could contribute to determining the relative importance of these sources in public health in the country.

## Data Availability

The original contributions presented in the study are included in the article/Supplementary material, further inquiries can be directed to the corresponding author.

## Footnotes

^1^ www.woah.org
