# Supplementary material for: Non-typhoidal Salmonella in food animals in Paraguay: predominant serovars and resistance phenotypes
Source: Front Vet Sci. 2025 Mar 25;12:1521469. doi: 10.3389/fvets.2025.1521469 (PMC11977416; doi:10.3389/fvets.2025.1521469)
Supplement: Supplementary file 1 [file Table_1.docx]

**Supplementary Table 1**. Number of isolates identified as susceptible (S), intermediate (I) or resistant (R) and percentage of resistant isolates depending on the host and serovar (color scale indicates level of resistance)

| **Host - Serovar** |  | Quinolones | | | | | | | | Betalactamics (Penicilins) | | | | | | | Betalactamics (3°G Cephalosporins) | | | | | | | | Aminoglycosides | | | | Miscellaneous | | | | Tetracyclines | | | | Sulfonamides | | | |
| --- | --- | --- | --- | --- | --- | --- | --- | --- | --- | --- | --- | --- | --- | --- | --- | --- | --- | --- | --- | --- | --- | --- | --- | --- | --- | --- | --- | --- | --- | --- | --- | --- | --- | --- | --- | --- | --- | --- | --- | --- |
|  | **N** | Nalidixic acid (NAL) | | | | Ciprofloxacin (CIP) | | | | Amoxicilin (AMX) | | | | Ampicilin (AMP) | | | Cefixime (CFM)^a^ | | | | Cefotaxime (CTX) | | | | Gentamicin (GEN) | | | | Nitrofurantoin (NIT) | | | | Tetracycline (TET) | | | | Trimethoprim Sulfamethoxazole (SXT) ^b^ | | | |
|  |  | **S** | **I** | **R** | **%** | **S** | **I** | **R** | **%** | **S** | **I** | **R** | **%** | **S** | **R** | **%** | **S** | **I** | **R** | **%** | **S** | **I** | **R** | **%** | **S** | **I** | **R** | **%** | **S** | **I** | **R** | **%** | **S** | **I** | **R** | **%** | **S** | **I** | **R** | **%** |
| **Poultry** |  |  |  |  |  |  |  |  |  |  |  |  |  |  |  |  |  |  |  |  |  |  |  |  |  |  |  |  |  |  |  |  |  |  |  |  |  |  |  |  |
| *S.* Heidelberg | 75 | 1 | 0 | 74 | 98.7 | 42 | 28 | 5 | 44 | 67 | 1 | 7 | 10.7 | 67 | 8 | 10.7 | 67 | 0 | 8 | 10.7 | 67 | 0 | 8 | 10.7 | 74 | 0 | 1 | 1.3 | 33 | 16 | 26 | 56 | 1 | 0 | 74 | 98.7 | 69 | 0 | 6 | 8 |
| *S.* Alachua | 5 | 5 | 0 | 0 | 0 | 5 | 0 | 0 | 0 | 5 | 0 | 0 | 0 | 5 | 0 | 0 | 5 | 0 | 0 | 0 | 5 | 0 | 0 | 0 | 5 | 0 | 0 | 0 | 5 | 0 | 0 | 0 | 5 | 0 | 0 | 0 | 5 | 0 | 0 | 0 |
| *S.* Anatum | 3 | 0 | 0 | 3 | 100 | 0 | 3 | 0 | 100 | 0 | 0 | 3 | 100 | 0 | 3 | 100 | 0 | 0 | 0 | 100 | 0 | 0 | 3 | 100 | 3 | 0 | 0 | 0 | 3 | 0 | 0 | 0 | 0 | 0 | 3 | 100 | 3 | 0 | 0 | 0 |
| *S.* Sandiego | 3 | 0 | 0 | 3 | 100 | 0 | 3 | 0 | 100 | 3 | 0 | 0 | 0 | 3 | 0 | 0 | 3 | 0 | 0 | 0 | 3 | 0 | 0 | 0 | 3 | 0 | 0 | 0 | 1 | 2 | 0 | 66.7 | 0 | 0 | 3 | 100 | 2 | 0 | 1 | 33.3 |
| *S.* Tennessee | 3 | 3 | 0 | 0 | 0 | 3 | 0 | 0 | 0 | 3 | 0 | 0 | 0 | 3 | 0 | 0 | 3 | 0 | 0 | 0 | 3 | 0 | 0 | 0 | 3 | 0 | 0 | 0 | 3 | 0 | 0 | 0 | 3 | 0 | 0 | 0 | 3 | 0 | 0 | 0 |
| *S.* Newport | 1 | 1 | 0 | 0 | 0 | 1 | 0 | 0 | 0 | 1 | 0 | 0 | 0 | 1 | 0 | 0 | 1 | 0 | 0 | 0 | 1 | 0 | 0 | 0 | 1 | 0 | 0 | 0 | 1 | 0 | 0 | 0 | 1 | 0 | 0 | 0 | 1 | 0 | 0 | 0 |
| *S.* Javiana | 1 | 0 | 0 | 1 | 100 | 0 | 1 | 0 | 100 | 1 | 0 | 0 | 0 | 1 | 0 | 0 | 1 | 0 | 0 | 0 | 1 | 0 | 0 | 0 | 1 | 0 | 0 | 0 | 0 | 1 | 0 | 100 | 0 | 0 | 1 | 100 | 1 | 0 | 0 | 0 |
| **Total Poultry** | **91** | **10** | **0** | **81** | **89** | **51** | **35** | **5** | **5.5** | **80** | **1** | **10** | **11** | **80** | **11** | **12** | **80** | **0** | **11** | **12** | **80** | **0** | **11** | **12.1** | **90** | **0** | **1** | **1.1** | **46** | **19** | **26** | **29** | **10** | **0** | **81** | **89** | **84** | **0** | **7** | **7.7** |
| **Swine** |  |  |  |  |  |  |  |  |  |  |  |  |  |  |  |  |  |  |  |  |  |  |  |  |  |  |  |  |  |  |  |  |  |  |  |  |  |  |  |  |
| *S.* Panama | 25 | 1 | 19 | 5 | 20 | 24 | 0 | 1 | 4 | 3 | 16 | 6 | 88 | 3 | 22 | 100 | 24 | 0 | 1 | 4 | 24 | 0 | 1 | 4 | 23 | 0 | 2 | 8 | 18 | 4 | 3 | 12 | 3 | 0 | 22 | 88 | 22 | 0 | 3 | 12 |
| *S.* Typhimurium | 20 | 1 | 5 | 14 | 70 | 18 | 1 | 1 | 5 | 2 | 10 | 8 | 40 | 2 | 18 | 90 | 19 | 0 | 0 | 0 | 20 | 0 | 0 | 0 | 17 | 0 | 3 | 14.3 | 7 | 5 | 8 | 40 | 4 | 0 | 16 | 76.2 | 17 | 1 | 1 | 5 |
| *S.* Anatum | 2 | 0 | 0 | 2 | 100 | 2 | 0 | 0 | 0 | 0 | 0 | 2 | 100 | 0 | 2 | 100 | 0 | 0 | 0 | 0 | 2 | 0 | 0 | 0 | 0 | 0 | 2 | 100 | 2 | 0 | 0 | 0 | 0 | 0 | 2 | 100 | 0 | 0 | 2 | 100 |
| *S.* Derby | 2 | 0 | 0 | 2 | 100 | 2 | 0 | 0 | 0 | 0 | 1 | 1 | 100 | 0 | 2 | 100 | 0 | 0 | 0 | 0 | 2 | 0 | 0 | 0 | 2 | 0 | 0 | 0 | 1 | 1 | 0 | 0 | 0 | 0 | 2 | 100 | 2 | 0 | 0 | 0 |
| Serogroup O7 | 3 | 1 | 1 | 1 | 33.3 | 3 | 0 | 0 | 0 | 1 | 2 | 0 | 0 | 1 | 2 | 66.8 | 3 | 0 | 0 | 0 | 3 | 0 | 0 | 0 | 3 | 0 | 0 | 0 | 2 | 0 | 1 | 33.3 | 1 | 0 | 2 | 100 | 3 | 0 | 0 | 0 |
| **Total Swine** | **52** | **3** | **25** | **24** | **46.1** | **49** | **1** | **2** | **3.8** | **6** | **29** | **17** | **32.6** | **6** | **46** | **88.5** | **46** | **0** | **1** | **2.1*** | **51** | **0** | **1** | **1.9** | **45** | **0** | **7** | **13.4** | **30** | **10** | **12** | **23** | **8** | **0** | **44** | **84.6** | **44** | **1** | **6** | **11.8*** |
| ^a^ Cefixime: total 47/52; five samples were not tested in pigs | | | | | | | | | | | | |  |  |  |  |  |  |  |  |  |  |  |  |  |  |  |  |  |  |  |  |  |  |  |  |  |  |  |  |
| ^b^ Trimethopim Sulfamethazole: 51/52; one sample not tested in pigs | | | | | | | | | | | | | |  |  |  |  |  |  |  |  |  |  |  |  |  |  |  |  |  |  |  |  |  |  |  |  |  |  |  |
